# Value of serum cytokine biomarkers TNF-α, IL-4, sIL-2R and IFN-γ for use in monitoring bacterial load and anti-tuberculosis treatment progress

**DOI:** 10.1016/j.cytox.2020.100028

**Published:** 2020-05-13

**Authors:** Wenjuan Nie, Jun Wang, Wei Jing, Wenhui Shi, Qingfeng Wang, Xuerui Huang, Baoyun Cai, Qiping Ge, Lihui Nie, Xiqin Han, Yadong Du, Jing Wang, Ru Guo, Naihui Chu

**Affiliations:** Tuberculosis Department, Beijing Chest Hospital Affiliated to Capital Medical University, No 9, Beiguan Street, Tongzhou District, Beijing 101149, PR China

**Keywords:** Serum cytokine, Biomarker, TNF-α, IL-4, sIL-2R, IFN-γ, Tuberculosis

## Abstract

Tuberculosis (TB) patient serum cytokine levels may be predictive of anti-tuberculosis treatment progress. Here, serum levels of cytokines TNF-α, IL-4, sIL-2R and IFN-γ were measured then correlated to clinical TB manifestations, bacterial burden, chest imaging findings and clinical course. Study subjects included 67 newly diagnosed pulmonary TB (PTB) patients with active disease admitted to Beijing Chest Hospital for anti-TB chemotherapeutic treatment. Blood was drawn at 0 months (pre-treatment), 1–2 months (at any time between 1 and 2 month) and after 6 months completion of treatment and serum TNF-α, IL-4, sIL-2R and IFN-γ levels were measured in duplicate using enzyme-linked immunosorbent assays (ELISAs). Correlation analysis was conducted to evaluate sensitivity and specificity of cytokine levels as predictors of disease activity and treatment progress. The results indicated that the pre-treatment serum TNF-α level of the smear-negative group was lower than that of the smear 1+ group, while serum TNF-α after 6 months completion of treatment and IFN-γ levels at 1–2 months and after 6 months completion of treatment were significantly lower, respectively, than at 0 months (before treatment) (P < 0.05). Using a cut-off value of 845 pg/ml, serum TNF-α level was predictive of treatment progress, with a sensitivity of 51%, specificity of 60% and AUC of 0.594 (P = 0.013). Meanwhile, using a cut-off value of 393 pg/ml, serum IFN-γ provided superior monitoring efficacy, with a sensitivity of 60%, specificity of 64% and AUC of 0.651 (P = 0.017). In conclusion, both serum TNF-α and IFN-γ levels might be useful biomarkers for monitoring treatment progress.

## Highlights

• Serum cytokine levels may correlate with tuberculosis patient disease status.
• Serum TNF-α level may be a useful biomarker for predicting bacillar burden.
• Serum TNF-α and IFN-γ levels may reliably monitor anti-TB treatment progress.
• Serum IL-4 level had no value while serum IL-2R level value awaits further study.
• Cytokine biomarkers are useful biomarkers in clinical TB patient care settings.

## 1 Introduction

Tuberculosis (TB), caused by *Mycobacterium tuberculosis* (MTB) infection, is a chronic disease that triggers the host cellular immune response. Although effective anti-TB agents are available, TB is still the most burdensome infectious disease in many areas of the world [1]. The host T cell-mediated immune response plays an important role in TB pathogenesis, while also conferring immunological protection against mycobacteria [2], [3], [4]. Notably, immune system activation by mycobacterial antigens is associated with elevated cytokine levels. Consequently, general inflammatory markers, such as erythrocyte sedimentation rate and C-reactive protein, have been used clinically in conjunction with chest roentgenogram imaging and bacterial burden testing to monitor TB disease activity [5]. However, such biomarkers offer unacceptably low specificity that is frequently affected by patient health status and inflammation.

According to several previous studies, analysis of levels of cytokines, such as TNF-α, IL-4, sIL-2R and IFN-γ, may provide a basis for the development of very valuable non-invasive methods for investigating in vivo states of immune activation in diseased and healthy individuals [6], [7], [8], [9], [10], [11], [12], [13], [14], [15], [16], [17]. Nevertheless, the value of these methods is controversial, since to date numerous studies evaluating such methods have failed to yield significant results to justify the use in clinical settings [18], [19], [20], [21], [22], [23], [24]. Furthermore, most such studies have focused on serum cytokine levels that are readily obtainable from blood samples, as opposed to methods involving in vitro TB antigenic stimulation of peripheral blood mononuclear cells requiring more arduous cell isolation steps. In this study, we measured levels of several cytokines in sera of patients with active PTB. Serum cytokine levels were then correlated to clinical manifestation, bacterial burden, chest imaging result and clinical course to determine their value when used for monitoring of TB disease activity and anti-TB treatment progress.

## 2. Method

### 2.1 Study population

During the course of this study, all procedures involving human participants were approved by the Ethics Committee of Beijing Chest Hospital, a hospital affiliated with Capital Medical University. Patients with newly diagnosed active PTB who were hospitalised at Beijing Chest Hospital were enrolled in this study. Patients were considered to have active PTB based on a positive culture for *Mycobacterium tuberculosis* (MTB), positive disease status confirmed via drug susceptibility testing (DST) and chest radiograph changes suggestive of TB infection. Patients with immunosuppressive risk factors or other characteristics were excluded: (1) age > 65 years; (2) previous anti-TB therapy for > 30 days; (3) treatment with glucocorticoid; (4) concomitant immune disorder; (5) concomitant diabetes; (6) infection with drug-resistant MTB.

### 2.2 Patient follow-up

Patient monitoring began just prior to treatment then continued throughout the six-month treatment period. Patients were treated with anti-TB agents (combinations of isoniazid, rifampicin, ethambutol and pyrazinamide). All patients were hospitalised during the first two weeks to ensure treatment compliance and were required to return to the hospital at least monthly. Symptom-positive patients exhibited respiratory symptoms that included fever, cough, sputa and/or malaise. Sputum smears and cultures were collected and examined every month from treatment start to treatment end and graded for bacterial burden as negative, 1+, 2+, 3 + and 4 + . Mandatory chest computed tomography (CT) scanning was performed at least every three months, with chest CT images showing presence of cavitating lesions interpreted as a positive result. Patients with side effects were asked to return to the hospital every half-month, weekly or were hospitalised according to disease severity stage.

### 2.3 Cytokine analysis

Blood samples were obtained prior to the start of chemotherapy then again between 1 and 2 months of chemotherapeutic treatment and again after 6 months completion of treatment. Each sample was collected in a 2-ml glass tube and serum was separated from clotted whole blood after centrifugation at 800 × *g* for 10 min. Processed samples were stored at −70 °C until use, with repeated freeze–thaw cycles avoided. Quantitation of serum cytokine level was performed in duplicate using enzyme-linked immunosorbent assay (ELISA) kits to detect cytokines TNF-α, IL-4, sIL-2R and IFN-γ (BioLegend, USA) based on instructions provided by the manufacturer. Adherent cell supernatants or standards were added to anti-cytokine monoclonal antibody-coated microtitre plates in duplicate, then enzyme-conjugated anti-cytokine monoclonal antibody directed against a second epitope of the cytokine molecule was added. After incubation and washing to remove unbound antibody, substrate was added then absorbance values at 490 nm were read. Standard curves were prepared from ELISAs conducted using cytokine standards and were used to quantify unknown cytokine levels in experimental samples.

### 2.4 Statistical method

Cytokine levels were calculated as the mean ± standard deviation (SD). Correlations between different variables (symptoms, image result, smear result and treatment time) with cytokine levels were tested via Spearman analysis using SPSS software. The statistics analysis of smear grade data was by linear trend test, and the difference between pre-treatment vs post-treatment was Wilcoxon signed-rank test. P values < 0.05 were considered statistically significant.

## 3. Result

### 3.1 Subject characteristics

Ultimately, 67 TB patients were enrolled in this study. All TB culture samples obtained from the 67 patients converted from positive to negative during the first two months of treatment; all patients were successfully cured by treatment completion. All patient characteristics, including gender, age, symptom, image finding, smear level and IGRA result are shown in Table 1, with distributions of variables shown, including patient ID, gender, age, sputum smear, symptom, image (chest CT) and IGRA positive rate. Patients with respiratory symptoms, such as fever, cough, sputa, or malaise, were graded symptom-positive. Bacterial burden was graded from negative to 4 + according to sputum smear bacterial numbers observed. Cytokine levels for each patient before treatment (0 month), at 1–2 months of treatment and after 6 months completion of treatment are shown in Supplemental Table 1.

**Table 1:** Demographic and clinical characteristics of the subjects.

Characteristics		Percentage (case/total)
Gender	Female	(35.8%) 24/67
	Male	(64.2%) 43/67
Age	＜50	(26.9%) 18/67
	≥50	(73.1%) 49/67
Symptom	Negative	(53.7%) 36/67
	Positive	(46.3%) 31/67
Image	Cavity negative	(55.2%) 37/67
	Cavity positive	(44.8%) 30/67
Smear	0+	(6.0%) 4/67
	1+	(38.8%) 26/67
	2+	(34.3%) 23/67
	3+	(14.9%) 10/67
	4+	(6.0%) 4/67
IGRA positive		(97.0%) 65/67

### 3.2 Correlation coefficient analysis between different variables and cytokine levels before treatment:

Several variables, including symptom, image result, smear result and treatment time were evaluated based on comparisons to serum cytokine levels of TNF-α, IL-4, sIL-2R and IFN-γ before treatment. Bivariate correlations were analyzed between a given cytokine value and different variables via Spearman analysis using SPSS software (Table 2). Correlations between TNF-α and smear result (correlation coefficient value = 0.209), TNF-α and treatment time (correlation coefficient value = −0.219), IFN-γ and smear result (correlation coefficient value = 0.285) and IFN-γ and treatment time (correlation coefficient value = −0.197) were all weak (P < 0.05). No associations were found between the other variables and serum cytokine levels.

**Table 2:** The correlation coefficient between serum cytokine levels (TNF-α, IL-4, sIL-2R and INF-γ) and different variables (symptom, image, smear and treatment) by Spearman analysis. The correlation coefficient between TNF-α and smear (correlation coefficient = 0.209, P = 0.003), TNF-α and treatment time (correlation coefficient = -0.219, P = 0.002), INF-γ and smear (correlation coefficient = 0.285, P = 0.000), INF-γ and treatment time (correlation coefficient = -0.197, P = 0.005) showed week correlation (P＜0.05).

	**TNF-α**		**IL-4**		**sIL-2R**		**INF-γ**	
	Correlation coefficient	P value	Correlation coefficient	P value	Correlation coefficient	P value	Correlation coefficient	P value
**Symptom**	–	0.650	–	0.135	–	0.970	–	0.369
**Image**	–	0.644	–	0.492	–	0.813	–	0.335
**Smear**	0.209	0.003	–	0.257	–	0.195	0.285	0.000
**Treatment time**	−0.219	0.002	–	0.545	–	0.254	−0.197	0.005

### 3.3 Serum cytokine levels according to AFB (acid-fast bacillus) smear grade at diagnosis

**Fig. 1:**
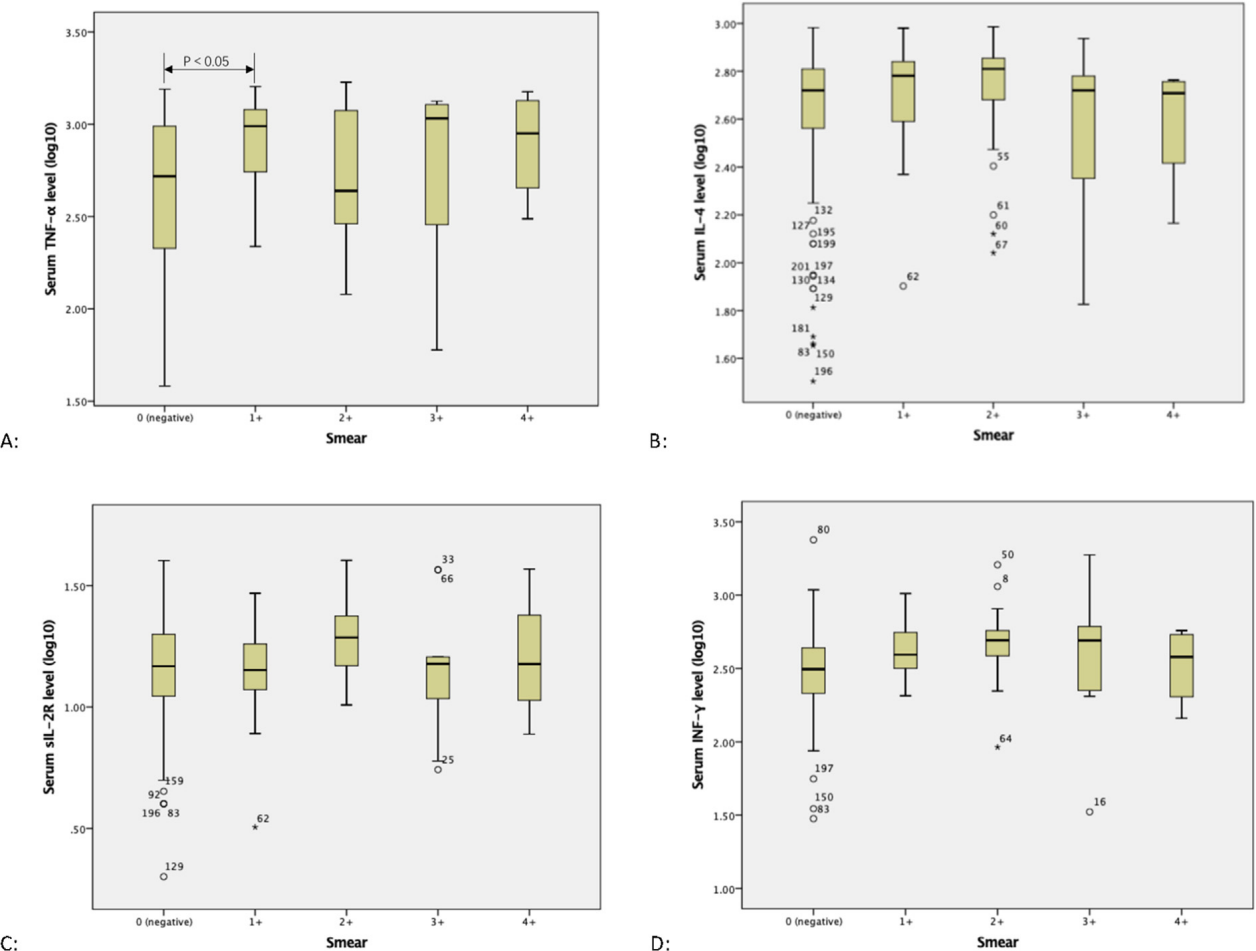
The statistics analysis of smear grade data was by linear trend test, with only TNF-α showed significant difference. Fig. 1A: The initial serum TNF-α levels in 0 month differed significantly from the level in 1–2 month (P < 0.05). There was no significant difference between other treatment time (P > 0.05). Fig. 1B: The initial serum IL-4 levels showed no significant difference between each group (P > 0.05). Fig. 1C: The initial serum sIL-2R levels showed no significant difference between each group (P > 0.05). Fig. 1D: The initial serum INF-γ levels showed no significant difference between each group (P > 0.05).

Serum TNF-α level differed depending on the acid-fast bacillus (AFB) smear grade. In Fig. 1A, initial serum TNF-α levels at 0 month differed significantly from levels measured at 1–2 months (P < 0.05), although no significant differences were observed (Fig. 1B, 1C, 1D) for other treatment times (P > 0.05).

Serum IL-4, sIL-2R and IFN-γ levels did not differ significantly according to AFB smear grade at the time of diagnosis (P > 0.05).

### 3.4 Longitudinal analysis of serum cytokine levels in patients with active TB who were monitored until therapy completion

**Fig. 2:**
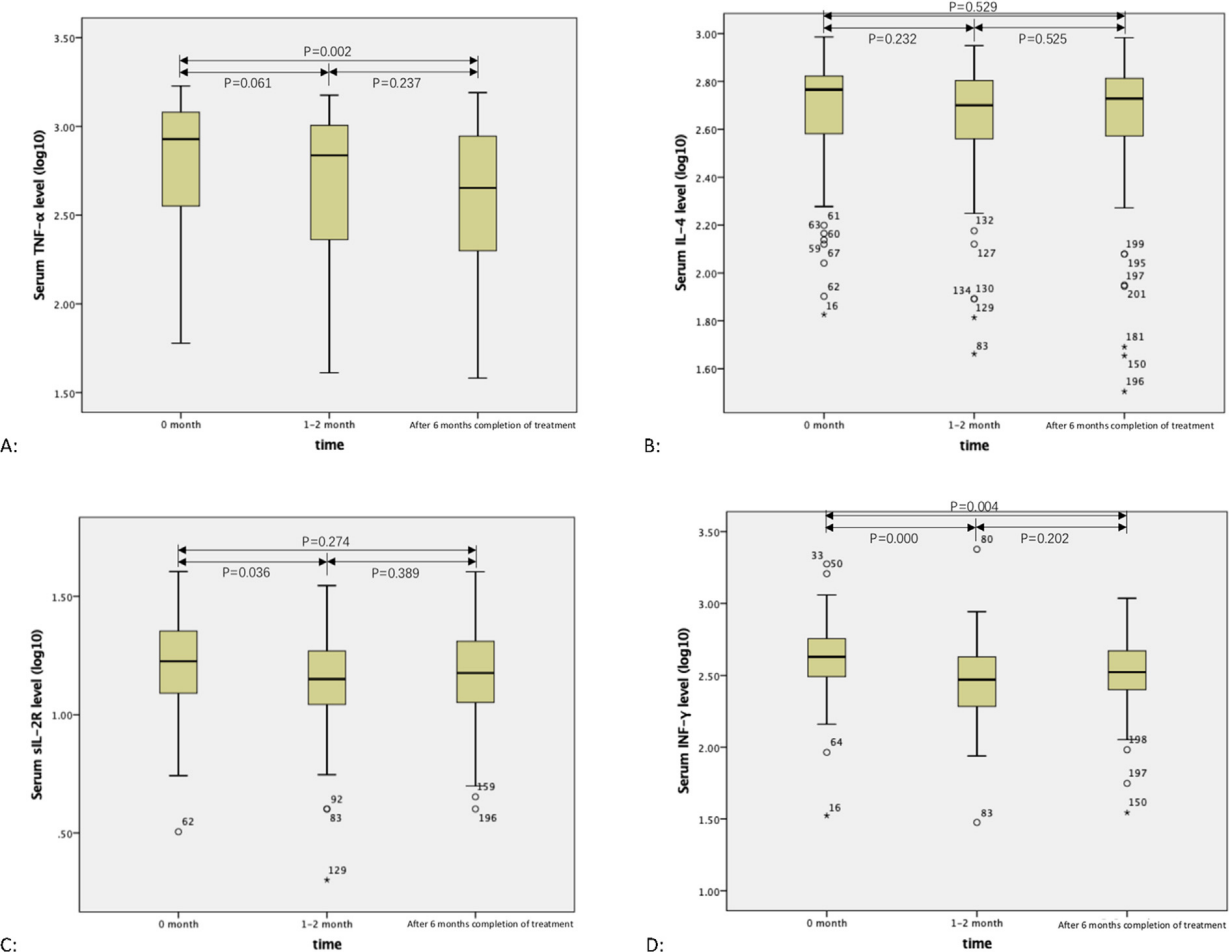
Longitudinal analysis of serum TNF-α, IL-4, sIL-2R and INF-γ levels in patients with active tuberculosis who were followed before and during treatment. Statistical analysis was compared between 0 month, 1–2 month and after 6 months completion of treatment. The P value below 0.05 was considered for significant difference. Fig. 1A: TNF-α shows significant decrease after 6 months completion of treatment when compared with 0 month (P = 0.002). No significant difference appeared in other group (P > 0.05). Fig. 1B: IL-4 shows no significant difference after compared with each group before or during the treatment (P > 0.05). Fig. 1C: sIL-2R shows significant decrease in 1–2 month when compared with 0 month (P = 0.036). No significant difference appeared in other group (P > 0.05). Fig. 1D: INF-γ shows significant decrease in 1–2 month when compared with 0 month (P = 0.000), and after 6 months completion of treatment when compared with 0 month (P = 0.004). No significant difference appeared in other group (P > 0.05).

As shown in Fig. 2A, significant changes were observed when serum TNF-α levels at the time of diagnosis (median: 848 pg/ml; interquartile range [IQR], 345–1201) were compared to levels at 1–2 months of treatment (median: 686 pg/ml; interquartile range [IQR], 226–1030) and after 6 months completion of treatment (median: 460 pg/ml; IQR, 189–891). Serum TNF-α levels after 6 months completion of treatment showed significant decreases compared with levels at the time of diagnosis (P = 0.002); however, no significant difference was observed when TNF-α levels at 1–2 months were compared with levels at the time of diagnosis, or when TNF-α levels after 6 months completion of treatment were compared with levels at 1–2 months of treatment.

**Fig. 3:**
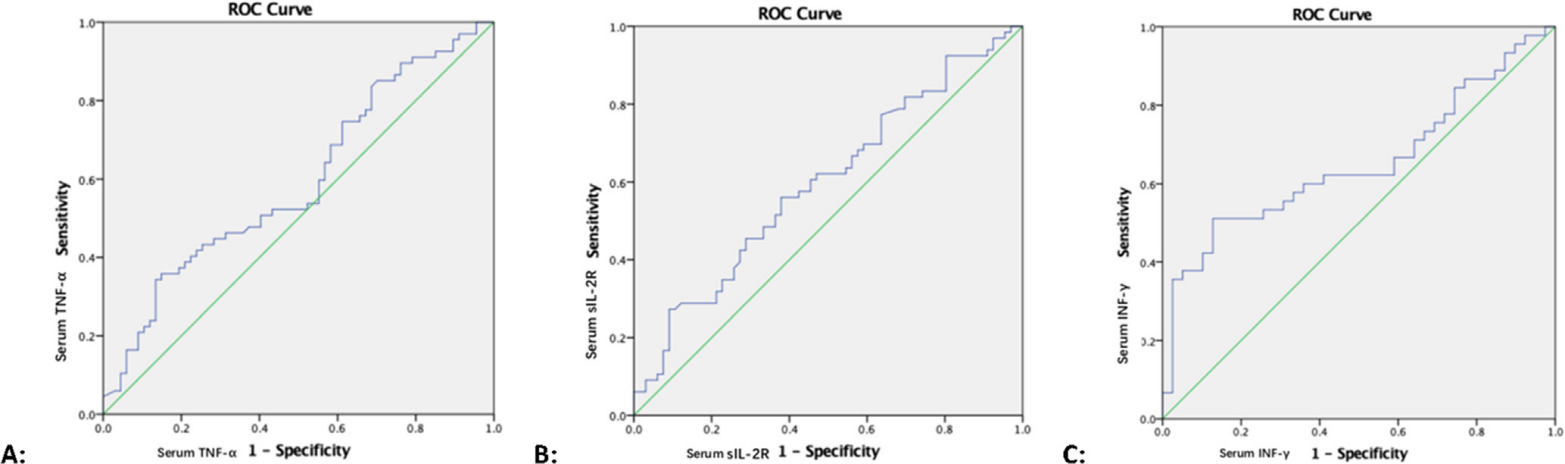
The receiver operating characteristic curve (ROC) analysis of serum TNF-α, IL-4, sIL-2R and INF-γ levels comparing the baseline value, the value in 1–2 month and after 6 months completion of treatment. Fig. 2A: For TNF-α shows significant decrease after 6 months completion of treatment when compared with 0 month (P = 0.002), the ROC analysis of serum TNF-α was performed by comparing the baseline value and the value after 6 months completion of treatment. Fig. 2B: For sIL-2R shows significant decrease in 1–2 month when compared with 0 month (P = 0.017), the ROC analysis of serum sIL-2R was performed by comparing the baseline value and the value in 1–2 month. Fig. 2C: For INF-γ shows significant decrease in 1–2 month when compared with 0 month (P = 0.002), the ROC analysis of serum INF-γ was performed by comparing the baseline value and the value in 1–2 month.

To evaluate the value of serum TNF-α level for use in monitoring TB treatment progress, we performed receiver operating characteristic curve (ROC) analysis-based comparisons of baseline (0 month) serum TNF-α level values and values after 6 months completion of treatment (Fig. 3A). Cut-off points were selected to maximise the sum of sensitivity and specificity. The cut-off point for optimal performance was 845 pg/ml for serum TNF-α (sensitivity: 51%; specificity: 60%; area under the curve: 0.594; P = 0.061). Using this cut-off value, 34 of 67 subjects (51%) scored positive for serum TNF-α at the time of TB diagnosis (Table 3). After 6 months completion of treatment, 19 (28%) of subjects became negative, attaining a lower positivity rate of disease than observed at the time of initial TB diagnosis (P = 0.013).

**Table 3:** Serial responses of serum TNF-α, IL-4, sIL-2R and IFN-γ in patients with active TB according to the cut-off values. The positive rate in 1–2 month or after 6 months completion of treatment was compared with 0 month using *X^2^* analysis.

	Positive rate in 0 month	Positive rate in 1–2 month	Positive rate after 6 months completion of treatment	Cut-off (pg/ml)	P value
Serum TNF-α	51% (34/67)	–	28% (19/67)	845	0.013
Serum sIL-2R	61% (41/67)	51% (34/67)	–	15	0.296
Serum IFN-γ	64% (43/67)	21% (14/67)	–	393	0.000

As shown in Fig. 2B, decreasing trend with treatment duration were observed when comparing serum IL-4 levels from time of diagnosis (median: 589 pg/ml; interquartile range [IQR], 398–675) to levels at 1–2 months of treatment (median: 502 pg/ml; interquartile range [IQR], 365–638) and after 6 months completion of treatment (median: 530 pg/ml; IQR, 344–648). But, no significant differences were observed when IL-4 levels at 1–2 months were compared with IL-4 levels at the time of diagnosis (P = 0.232), or when levels after 6 months completion of treatment were compared with levels at the time of diagnosis (P = 0.529) or when levels after 6 months completion of treatment were compared with levels at 1–2 months of treatment (P = 0.525).

Due to the fact that we observed no significant serum IL-4 level difference before and during treatment, we did not perform ROC analysis based on serum IL-4 level.

As shown in Fig. 2C, significant changes were observed when comparing serum sIL-2R levels at the time of diagnosis (median: 16 pg/ml; interquartile range [IQR], 12–23), at 1–2 months of treatment (median: 14 pg/ml; interquartile range [IQR], 11–19) and after 6 months completion of treatment (median: 16 pg/ml; IQR, 11–21). Serum sIL-2R levels at 1–2 months of treatment were significantly lower than levels at the time of diagnosis (P = 0.036). However, no significant difference was observed between sIL-2R levels measured after 6 months completion of treatment versus the level at the time of diagnosis, or for levels after 6 months completion of treatment versus levels at 1–2 months of treatment.

To evaluate the predictive value of serum sIL-2R for TB treatment monitoring, we performed ROC analysis of serum sIL-2R levels by comparing baseline values to levels at 1–2 months (Fig. 3B) using cut-off points that maximised the sum of sensitivity and specificity. The cut-off point for optimal performance was 14 pg/ml for serum sIL-2R (sensitivity: 61%; specificity: 55%; area under the curve: 0.597; P = 0.054). Using this cut-off value, 41 of 67 subjects (61%) scored positive for serum sIL-2R at the time of TB diagnosis, while at 1–2 months of treatment, 34 (51%) subjects tested negative. However, although levels at 1–2 months were lower than levels at the time of diagnosis, this difference was not significant (P = 0.296).

As shown in Fig. 2D, significant changes were observed when comparing serum IFN-γ levels at the time of diagnosis (median: 490 pg/ml; interquartile range [IQR], 303–607) to levels at 1–2 months of treatment (median: 357 pg/ml; interquartile range [IQR], 273–431) and to levels after 6 months completion of treatment (median: 419 pg/ml; IQR, 252–511). Meanwhile, serum IFN-γ levels at 1–2 months exhibited a significant decrease relative to levels at the time of diagnosis (P = 0.000), and serum IFN-γ levels after 6 months completion of treatment exhibited a significant decrease relative to levels at the time of diagnosis (P = 0.004). No significant difference was observed when IFN-γ-α levels after 6 months completion of treatment were compared with respective levels at 1–2 months of treatment.

To evaluate the applicability of serum IFN-γ to TB treatment monitoring, we performed ROC analysis of serum IFN-γ by comparing baseline levels to respective values at 1–2 months (Fig. 3C) using cut-off points that maximised the sum of sensitivity and specificity. The cut-off point for optimal performance was 393 pg/ml for serum IFN-γ (sensitivity: 60%; specificity: 64%; area under the curve: 0.651; P = 0.017). Using this cut-off value, 43 of 67 subjects (64%) scored positive for serum IFN-γ at the time of TB diagnosis; at 1–2 months, 14 (21%) subjects tested negative, reflecting a reduced TB-positivity rate relative to the rate at the time of diagnosis (P = 0.000).

## 4. Discussion

TB is an inflammatory disease that is increasingly viewed as an unbalanced immune response to *M. tuberculosis* infection, with disease progression or containment determined mainly by the relative strength of the MTB invader versus that of the host defence system [25]. During disease processes, host cytokines act as messengers that help integrate immune system components during combat against invading pathogens [26]. As is already known, cytokine roles during the TB disease process are complex and multifaceted and are influenced by different host states [27]. Therefore, systematic and comprehensive studies are needed to explore cytokine activities and how they relate to *M. tuberculosis* infection. Furthermore, a better understanding of PTB patient immune changes during treatment is more urgently needed in order to provide a theoretical foundation to guide development of immunologically based diagnostic, therapeutic and monitoring tests of treatment progress. Current TB monitoring methods exploit the fact that TB can cause substantial tissue damage; therefore, pulmonary lesion progression is usually monitored as a measure of disease activity and/or severity. Here, analyses were conducted to compare serum cytokine levels as biomarker signatures among sub-groups of PTB patients stratified by AFB smear status, extent of radiographic disease and treatment duration. Specifically, levels of cytokines relevant to host immune responses to MTB infection were assessed as predictive biomarkers of disease activity and treatment success.

Type 1 cytokines, specifically TNF-α, are crucial for protection against *M. tuberculosis* infection [28]. In addition, TNF-α is important for containment of persistent infection and prevention of reactivation [29]. However, TNF-α has also been demonstrated to be a key participant in pathogenesis and disease progression [30]. Indeed, high TNF-α levels may actually promote granuloma dissemination, infection progression and increased pathology [31]. Further support for this concept was obtained in one study showing that incomplete anti-inflammatory treatment-based reduction of TNF-α levels improved TB outcomes [32]. Meanwhile, other data suggest that elevated TNF-α levels are secondary to disease severity, revealing an important association between TNF-α and both degree of TB pathology and disease extent; such results have revealed a direct correlation of TNF-α level with bacterial burden and a negative correlation with time to sputum culture conversion [6], [7]. However, results of another study indicated levels of TNF-α expression did not increase in TB patients relative to levels in healthy donors [18], suggesting that TNF-α level might not be related to bacterial burden [6]. In our study, although initial serum TNF-α levels showed weak correlations with smear bacillar load (correlation coefficient = 0.209, P = 0.003), with initial serum TNF-α level in the smear-negative group significantly lower than that of the smear 1 + group (P < 0.05), no significant associations between bacillar burden and serum TNF-α level at other treatment timepoints were observed. These results indicate that serum TNF-α might not be a biomarker indicative of bacillar burden at the time of patient diagnosis. Many studies have monitored the cytokine efficacy of anti-TB therapy. The most method was to monitor the progressive decrease before treatment and therapy completion [33], [34], [35], which measured the cytokine response after treatment compared with the baseline value. A significant decrease might be the evidence of a potential biomarker for monitoring TB treatment. In fact, here serum TNF-α levels actually decreased significantly after 6 months completion of treatment as compared with the level at 0 month for cases achieving treatment success (P = 0.002). Thus, when these results are considered together, serum TNF-α might serve as a marker of both bacillar burden or of TB treatment efficacy. The cut-off values obtained from ROC curves might also be analyzed for the biomarker evaluation [33]. Analysis of ROC results and cut-off values here indicated that at the cut-off value of 845 pg/ml, TNF-α served as a potential biomarker for treatment efficacy monitoring, with a sensitivity of 51%, specificity of 60% and AUC of 0.594 (P = 0.013).

IL-4 is known as a regulatory cytokine that participates in control of the immune response against infection [36]. In addition, it has a role in the development of immunopathology through its regulation of TNF-α activity during progressive disease. Indeed, IL-4 produced in response to MTB infection may down-regulate the immune response to limit tissue injury; however, excessive production of this cytokine may result in failure to control infection [37], thus highlighting the controversy surrounding the role of IL-4 during TB progression. Moreover, several previous studies have demonstrated increased IL-4 production in human TB patients, especially those with cavitary disease [8], [9], while in other studies, IL-4 either promotes TB progression or assumes a merely passive role as a marker of disease activity [10], [11]. Meanwhile, other studies show no detectable IL-4 level in any TB patient and no significant IL-4 level differences between TB patients and controls [19], [20], [21], [22], [23]. To further fuel the controversy, when IL-4 levels were evaluated in TB patients before and after therapy, some studies reported no statistically significant changes in IL-4 levels, while other studies reported increased IL-4 levels before and after treatment [38], [39]. In our study, although decreases in serum IL-4 levels were observed with treatment, no significant difference in IL-4 levels were observed when comparing different treatment times (P > 0.05). Therefore, our results do not support use of serum IL-4 level as a biomarker for monitoring of MTB burden and treatment efficacy.

The cytokine sIL-2R can be detected in supernatants of cultures of T-lymphocytes activated by mitogen or antigen [5], [40]. As TB is a disease regulated entirely by the host cell-mediated delayed-type immune response against *M. tuberculosis* [4], increased levels of sIL-2R in TB patients denote a substantial degree of T lymphocyte activation that likely influences the disease course [41]. Notably, previous studies have demonstrated that the extent and spread of pulmonary TB infection affected serum levels of sIL-2R; specifically, patients with extensive parenchymal involvement, as observed via chest roentgenogram, and high bacterial burdens had significantly higher levels of sIL-2R compared with patients with lesser lesions and low bacteria burden [1]. The mechanism underlying the occurrence of higher sIL-2R levels in sputum-positive patients likely reflects a response by a larger population of lymphocytes to abundant bacterial loads in that patient group relative to other patient groups [41]. However, in our study patients with serious lesions (determined via imaging) and high bacillar levels (determined via smear analysis) did not exhibit significantly higher sIL-2R levels as compared to patients with minor lesions and lower smear scores. By contrast, other studies have claimed that changes in sIL-2R serum level generally coincide with clinical course and response to anti-TB agents, with reduced values observed after treatment [1], [41]. Nonetheless, in our study a significant change in serum IL-2R level was observed between 0 and 1–2 months, but the result was not significant when analyzed using ROC and cut-off values. Therefore, more clinical research is needed to clearly demonstrate whether sIL-2R may serve as an effective biomarker for use in monitoring MTB infection status and treatment efficacy.

IFN-γ is a potential immunodiagnostic marker for use in detection of MTB infection, since it is released by activated CD4 + T cells as a key component of the protective anti-MTB immune response [42]. Nevertheless, conflicting results regarding the correlation between IFN-γ response magnitude and bacillar load have been reported, with some reports [12], [13], [14], [15], [16], [17] suggesting an association, while other studies revealed no such association [24]. In this study, although initial serum IFN-γ levels showed weak correlations with smear level (correlation coefficient = 0.285, P = 0.000), initial serum IFN-γ levels did not significantly differ from levels for different smear groups ranging from 0 to 4+ (P > 0.05); thus, serum IFN-γ level might not effectively reflect bacillar burden at the time of diagnosis. Conversely, at the time of treatment completion, numerous IFN-γ studies have supported its use for effectively monitoring anti-TB therapeutic efficacy, with some studies demonstrating a progressive decrease in serum IFN-γ level at therapeutic completion [43], [44], [45]. By contrast, other studies have shown no change or only a minimal change in IFN-γ level after successful treatment [46], [47], [48]. Here, serum IFN-γ decreased significantly during initial treatment between 0 and to 1–2 months for cases ultimately achieving treatment success (P = 0.000), and significant difference in IFN-γ level observed between after 6 months completion of treatment and 0 month. Therefore, according to the previous method [33], [34], [35], in PTB patients, serum IFN-γ level might also be a useful biomarker for monitoring of anti-TB treatment efficacy, but not for monitoring of bacillar burden. ROC analysis using a cut-off value of 393 pg/ml indicated that serum IFN-γ level possessed superior value as a biomarker of treatment progress, with a sensitivity of 60%, specificity of 64% and AUC of 0.651 (P = 0.017), compared with TNF-α in this study.

In this study, some cytokines were dynamically changed differently during the treatment. For instance, TNF-α levels showed a continuous downward trend from TB diagnosis to TB treatment completion. But other cytokines such as IFN-γ level increased after TB treatment. These inconsistencies could be observed in other studies. For instantce, IFN-γ was shown to increase during treatment in Bai study [36], which was also consistent with the results of Sahiratmadja [49] and Larissa [50]. However, in other studies, untreated individuals presented with much higher levels of this cytokine, which gradually decreased during treatment [51]. These inconsistencies may be because the patients enrolled were in a different status of infection and treatment and the techniques and means of detecting cytokines were also different. In addition, these results indicated that some immune function including IFN-γ might recover in some way in these cured patients after 6 months of comprehensive treatment.

In summary, the results of this study demonstrate that in PTB patients serum cytokine including TNF-α, IL-4, sIL-2R and IFN-γ level might not serve as a useful biomarker for predicting bacillar burden, while serum TNF-α and IFN-γ levels both may be useful tools for monitoring anti-TB chemotherapeutic treatment response. Therefore, these cytokine biomarkers have potential value for use in clinical PTB patient care management.

## CRediT author statement

**Wenjuan Nie**: Writing - Reviewing and Editing, Conceptualization, Methodology, Software. Jun Wang: Data curation, Writing - Original draft preparation. **Wei Jing**, **Wenhui Shi**: Lab Operation. **Qingfeng Wang**, **Xuerui Huang**, **Baoyun Cai**, **Qiping Ge**, **Lihui Nie**, **Xiqin Han**, **Yadong Du**, **Jing Wang**, **Ru Guo**: Patients Enrolling. **Naihui Chu**: Supervision, Funding, Reviewing and Editing.

## Declaration of Competing Interest

The authors declare that they have no known competing financial interests or personal relationships that could have appeared to influence the work reported in this paper.
