# Supplementary material for: Value of serum cytokine biomarkers TNF-α, IL-4, sIL-2R and IFN-γ for use in monitoring bacterial load and anti-tuberculosis treatment progress
Source: Cytokine X. 2020 May 13;2(2):100028. doi: 10.1016/j.cytox.2020.100028 (PMC7885884; doi:10.1016/j.cytox.2020.100028)
Supplement: Supplementary data 1 [file mmc1.docx]

**Supplement table 1:** Each patients’ characteristics and different serum cytokine levels during the treatment.

| ID | Gender | Age | Symptom  (1 means symptom positive, 0 means negative) | Image (1 means cavity positive; 0 means negative) | Smear | Cytokine levels in 0 month | | | | Cytokine levels in 1-2 month | | | | Cytokine levels after 6 months completion of treatment | | | |
| --- | --- | --- | --- | --- | --- | --- | --- | --- | --- | --- | --- | --- | --- | --- | --- | --- | --- |
|  |  |  |  |  |  | TNF-α | IL-4 | sIL-2R | INF-γ | TNF-α | IL-4 | sIL-2R | INF-γ | TNF-α | IL-4 | sIL-2R | INF-γ |
| 1 | Male | 21 | 0 | 1 | 1+ | 900.00 | 234.00 | 12.00 | 303.13 | 908.99 | 397.98 | 10.88 | 400.00 | 699.97 | 397.98 | 8.49 | 458.60 |
| 2 | Male | 63 | 1 | 1 | 2+ | 1300.00 | 521.95 | 10.31 | 648.99 | 1273.78 | 467.85 | 11.72 | 504.76 | 1000.00 | 564.00 | 8.00 | 497.00 |
| 3 | Male | 32 | 1 | 0 | 1+ | 1400.00 | 628.57 | 14.58 | 592.84 | 1203.16 | 758.92 | 18.19 | 334.86 | 900.00 | 467.00 | 9.00 | 424.00 |
| 4 | Female | 23 | 0 | 0 | 1+ | 1239.47 | 724.85 | 18.19 | 785.79 | 1071.37 | 622.06 | 14.11 | 508.09 | 1000.00 | 743.00 | 12.00 | 348.45 |
| 5 | Female | 55 | 1 | 1 | 1+ | 552.14 | 592.39 | 11.79 | 505.59 | 842.23 | 583.02 | 9.90 | 384.32 | 170.00 | 738.22 | 11.55 | 284.27 |
| 6 | Female | 36 | 1 | 0 | 2+ | 1296.91 | 441.05 | 16.14 | 665.27 | 1500.00 | 365.00 | 13.00 | 427.00 | 1100.00 | 654.00 | 11.00 | 535.00 |
| 7 | Male | 63 | 0 | 1 | 2+ | 1171.96 | 649.24 | 12.37 | 543.85 | 1029.88 | 465.22 | 14.52 | 637.90 | 800.00 | 532.00 | 14.00 | 528.00 |
| 8 | Male | 29 | 0 | 1 | 2+ | 1690.24 | 733.17 | 22.31 | 1145.28 | 1500.00 | 635.00 | 20.00 | 796.00 | 1300.00 | 643.00 | 22.00 | 953.00 |
| 9 | Female | 21 | 0 | 1 | 1+ | 1188.36 | 521.17 | 13.27 | 1025.76 | 891.99 | 538.68 | 9.27 | 459.51 | 195.00 | 671.81 | 20.89 | 448.57 |
| 10 | Female | 47 | 0 | 1 | 1+ | 1000.00 | 652.00 | 18.00 | 863.71 | 1242.53 | 645.90 | 13.16 | 643.00 | 1547.72 | 520.57 | 17.53 | 748.00 |
| 11 | Female | 33 | 1 | 1 | 1+ | 1200.69 | 692.50 | 18.66 | 632.21 | 1274.66 | 749.95 | 14.14 | 415.47 | 251.59 | 891.71 | 16.55 | 210.82 |
| 12 | Male | 47 | 0 | 1 | 4+ | 1500.00 | 563.00 | 37.00 | 572.80 | 1406.75 | 597.46 | 11.71 | 627.62 | 1036.46 | 590.43 | 35.64 | 430.83 |
| 13 | Female | 41 | 0 | 0 | 1+ | 1600.00 | 654.00 | 19.00 | 472.80 | 1319.24 | 713.98 | 13.34 | 2384.29 | 1167.08 | 641.39 | 16.59 | 497.00 |
| 14 | Male | 33 | 1 | 0 | 3+ | 1214.59 | 602.74 | 15.84 | 777.77 | 904.61 | 732.95 | 9.08 | 294.59 | 267.66 | 602.12 | 24.85 | 382.53 |
| 15 | Female | 29 | 0 | 0 | 2+ | 1200.00 | 696.88 | 17.95 | 807.26 | 975.83 | 729.58 | 19.06 | 436.61 | 700.00 | 654.00 | 18.00 | 378.48 |
| 16 | Male | 50 | 0 | 0 | 3+ | 60.00 | 67.00 | 6.00 | 33.29 | 41.48 | 45.89 | 4.00 | 29.88 | 38.21 | 45.09 | 5.00 | 35.01 |
| 17 | Male | 27 | 1 | 1 | 2+ | 1250.19 | 556.36 | 13.11 | 556.34 | 1235.00 | 467.24 | 11.94 | 458.36 | 1100.00 | 654.00 | 10.00 | 684.90 |
| 18 | Male | 26 | 1 | 0 | 4+ | 1200.00 | 580.00 | 14.68 | 505.85 | 1100.00 | 734.00 | 12.00 | 424.45 | 922.87 | 591.85 | 11.67 | 499.00 |
| 19 | Male | 29 | 1 | 0 | 1+ | 1178.55 | 956.51 | 17.40 | 674.60 | 900.00 | 837.00 | 15.00 | 467.00 | 1100.00 | 923.00 | 14.00 | 532.00 |
| 20 | Male | 22 | 0 | 0 | 1+ | 800.00 | 389.72 | 7.78 | 286.14 | 764.21 | 501.76 | 7.06 | 180.72 | 295.89 | 691.45 | 19.94 | 293.16 |
| 21 | Male | 61 | 1 | 0 | 3+ | 1113.89 | 569.49 | 14.68 | 610.63 | 1109.24 | 432.97 | 14.57 | 404.55 | 780.00 | 564.00 | 10.13 | 532.22 |
| 22 | Male | 18 | 1 | 0 | 1+ | 1077.87 | 617.18 | 13.36 | 292.30 | 1055.40 | 540.87 | 14.79 | 432.95 | 150.20 | 724.68 | 24.68 | 402.99 |
| 23 | Female | 26 | 1 | 0 | - | 1102.44 | 593.77 | 19.27 | 466.74 | 999.00 | 461.00 | 16.00 | 256.00 | 888.00 | 645.00 | 14.00 | 367.00 |
| 24 | Female | 28 | 1 | 1 | 2+ | 1064.08 | 547.39 | 10.20 | 384.26 | 978.44 | 507.03 | 12.95 | 461.04 | 203.18 | 634.77 | 30.03 | 205.92 |
| 25 | Male | 46 | 0 | 1 | 3+ | 320.18 | 224.81 | 5.53 | 223.58 | 200.00 | 287.00 | 4.00 | 178.00 | 150.00 | 456.00 | 4.50 | 276.00 |
| 26 | Male | 38 | 0 | 1 | 1+ | 848.07 | 477.32 | 14.91 | 376.68 | 600.00 | 590.00 | 11.00 | 212.00 | 500.00 | 602.00 | 8.00 | 321.00 |
| 27 | Male | 36 | 1 | 0 | 1+ | 1243.95 | 617.64 | 11.05 | 512.13 | 891.71 | 528.43 | 14.90 | 431.36 | 1200.00 | 591.00 | 9.00 | 438.09 |
| 28 | Male | 44 | 1 | 0 | 1+ | 968.58 | 470.97 | 13.85 | 396.18 | 807.92 | 461.09 | 9.25 | 289.35 | 800.00 | 527.00 | 6.00 | 278.00 |
| 29 | Female | 30 | 1 | 1 | 1+ | 508.35 | 301.81 | 8.30 | 205.96 | 584.27 | 315.90 | 5.58 | 181.78 | 46.71 | 535.01 | 16.45 | 227.59 |
| 30 | Male | 63 | 0 | 0 | 3+ | 1278.10 | 583.31 | 15.46 | 480.11 | 1100.00 | 638.00 | 12.00 | 321.00 | 876.00 | 512.00 | 13.00 | 478.00 |
| 31 | Female | 61 | 0 | 0 | 1+ | 864.10 | 373.55 | 10.05 | 366.74 | 687.44 | 456.77 | 9.75 | 236.35 | 754.00 | 487.00 | 8.00 | 305.47 |
| 32 | Female | 26 | 0 | 1 | 3+ | 1324.00 | 422.72 | 12.17 | 501.13 | 938.07 | 458.52 | 11.10 | 389.98 | 821.70 | 345.39 | 12.88 | 550.11 |
| 33 | Male | 26 | 0 | 1 | 3+ | 1040.25 | 628.11 | 36.80 | 1882.69 | 783.00 | 498.00 | 31.00 | 854.00 | 853.00 | 571.00 | 29.00 | 1065.00 |
| 34 | Female | 30 | 0 | 0 | 1+ | 1543.00 | 345.39 | 10.47 | 340.01 | 1082.46 | 345.39 | 11.19 | 324.38 | 1232.06 | 590.14 | 11.55 | 635.73 |
| 35 | Female | 24 | 1 | 0 | 1+ | 985.43 | 541.95 | 12.31 | 387.75 | 765.00 | 487.00 | 12.00 | 219.00 | 799.00 | 612.00 | 11.00 | 378.00 |
| 36 | Male | 47 | 0 | 0 | 4+ | 664.30 | 463.80 | 7.74 | 283.56 | 456.00 | 339.96 | 9.28 | 187.82 | 420.12 | 461.00 | 10.13 | 333.78 |
| 37 | Male | 32 | 1 | 1 | 3+ | 1333.82 | 863.81 | 16.08 | 577.59 | 678.00 | 765.00 | 14.00 | 321.00 | 843.00 | 719.00 | 12.70 | 498.00 |
| 38 | Male | 61 | 1 | 0 | 1+ | 1084.19 | 591.95 | 12.23 | 420.04 | 686.00 | 409.00 | 11.00 | 213.00 | 977.00 | 367.00 | 10.00 | 378.00 |
| 39 | Female | 25 | 0 | 1 | 2+ | 1299.93 | 585.50 | 13.58 | 387.14 | 1098.00 | 561.00 | 11.00 | 278.00 | 1183.00 | 491.00 | 12.00 | 215.00 |
| 40 | Male | 64 | 1 | 0 | 1+ | 1260.42 | 724.41 | 16.95 | 556.41 | 987.00 | 671.00 | 13.00 | 327.00 | 1043.00 | 842.00 | 14.00 | 437.00 |
| 41 | Male | 46 | 1 | 0 | 2+ | 123.81 | 811.86 | 23.38 | 493.12 | 366.11 | 615.37 | 19.64 | 294.27 | 536.09 | 645.57 | 19.73 | 290.54 |
| 42 | Male | 54 | 0 | 1 | 2+ | 572.70 | 750.14 | 25.00 | 581.76 | 202.96 | 611.62 | 14.68 | 388.42 | 172.72 | 379.08 | 16.53 | 247.93 |
| 43 | Male | 45 | 1 | 1 | 2+ | 517.64 | 827.21 | 27.17 | 564.47 | 363.00 | 720.00 | 18.06 | 278.00 | 487.00 | 790.00 | 16.00 | 176.00 |
| 44 | Male | 34 | 0 | 1 | 2+ | 178.00 | 645.73 | 19.35 | 541.32 | 79.38 | 521.76 | 28.17 | 380.35 | 161.68 | 862.33 | 35.81 | 295.91 |
| 45 | Male | 53 | 0 | 0 | 2+ | 408.92 | 862.33 | 25.82 | 532.76 | 508.53 | 614.68 | 26.40 | 404.40 | 134.15 | 341.04 | 40.11 | 519.12 |
| 46 | Male | 65 | 0 | 0 | 2+ | 370.09 | 669.60 | 26.33 | 480.49 | 40.91 | 645.46 | 14.93 | 238.51 | 332.34 | 266.23 | 19.05 | 338.22 |
| 47 | Male | 29 | 0 | 1 | - | 345.00 | 536.48 | 22.76 | 163.36 | 85.72 | 393.02 | 13.70 | 119.51 | 575.24 | 49.13 | 11.99 | 120.40 |
| 48 | Male | 54 | 0 | 0 | 1+ | 385.71 | 831.48 | 26.05 | 334.91 | 357.57 | 554.54 | 16.46 | 298.70 | 162.64 | 367.36 | 24.45 | 229.25 |
| 49 | Male | 40 | 1 | 1 | 2+ | 329.58 | 659.97 | 12.86 | 389.00 | 343.37 | 722.32 | 20.79 | 127.24 | 243.00 | 625.00 | 17.00 | 300.73 |
| 50 | Male | 60 | 0 | 0 | 2+ | 432.94 | 968.20 | 19.98 | 1611.04 | 234.00 | 710.00 | 17.00 | 876.00 | 432.00 | 809.00 | 15.00 | 1087.00 |
| 51 | Female | 34 | 1 | 1 | 2+ | 223.94 | 603.32 | 18.14 | 406.34 | 201.82 | 598.99 | 16.21 | 278.00 | 149.95 | 676.58 | 12.36 | 112.90 |
| 52 | Male | 19 | 1 | 0 | 2+ | 254.00 | 676.58 | 24.08 | 382.99 | 126.72 | 360.46 | 35.07 | 212.17 | 115.31 | 446.08 | 32.11 | 301.66 |
| 53 | Female | 25 | 0 | 0 | 1+ | 316.00 | 702.23 | 23.57 | 322.51 | 213.00 | 782.00 | 17.00 | 167.00 | 145.00 | 514.00 | 19.00 | 389.00 |
| 54 | Male | 58 | 1 | 0 | 1+ | 443.82 | 762.68 | 29.41 | 426.19 | 254.00 | 890.00 | 21.00 | 214.00 | 367.00 | 961.00 | 26.00 | 376.00 |
| 55 | Female | 28 | 1 | 0 | 2+ | 366.56 | 253.50 | 40.23 | 278.96 | 235.49 | 361.93 | 21.76 | 195.78 | 254.00 | 486.00 | 38.66 | 158.72 |
| 56 | Female | 64 | 0 | 1 | 1+ | 463.00 | 626.92 | 21.76 | 275.30 | 136.27 | 307.20 | 21.00 | 295.73 | 364.00 | 287.00 | 18.00 | 178.00 |
| 57 | Male | 54 | 1 | 1 | - | 126.10 | 307.20 | 30.06 | 387.89 | 226.27 | 418.03 | 22.14 | 364.03 | 95.63 | 445.19 | 17.64 | 258.28 |
| 58 | Male | 22 | 0 | 0 | 1+ | 217.81 | 267.66 | 16.81 | 316.80 | 217.28 | 177.42 | 17.19 | 189.00 | 241.00 | 279.72 | 22.45 | 267.00 |
| 59 | Female | 34 | 0 | 0 | 3+ | 285.85 | 137.44 | 10.83 | 204.35 | 76.72 | 394.85 | 20.72 | 149.32 | 129.67 | 307.85 | 9.50 | 265.94 |
| 60 | Female | 23 | 0 | 0 | 2+ | 119.80 | 131.88 | 23.38 | 221.89 | 198.00 | 132.00 | 19.00 | 123.00 | 281.00 | 254.00 | 17.00 | 289.00 |
| 61 | Female | 27 | 0 | 1 | 2+ | 483.77 | 158.17 | 19.73 | 439.97 | 430.03 | 186.88 | 25.61 | 119.91 | 450.00 | 120.00 | 22.00 | 287.00 |
| 62 | Male | 20 | 0 | 1 | 1+ | 581.61 | 79.91 | 3.20 | 300.00 | 425.00 | 65.00 | 2.00 | 265.00 | 253.00 | 32.00 | 4.00 | 211.00 |
| 63 | Male | 54 | 1 | 0 | 4+ | 307.68 | 146.25 | 15.42 | 144.75 | 243.00 | 78.00 | 8.00 | 87.00 | 276.00 | 88.00 | 14.00 | 56.00 |
| 64 | Female | 31 | 0 | 0 | 2+ | 230.06 | 297.26 | 18.52 | 92.09 | 123.00 | 210.00 | 11.00 | 89.00 | 156.00 | 187.00 | 15.00 | 96.00 |
| 65 | Male | 56 | 1 | 1 | - | 206.26 | 189.42 | 26.01 | 247.05 | 143.00 | 150.00 | 20.00 | 167.00 | 345.00 | 120.00 | 29.00 | 212.00 |
| 66 | Male | 31 | 1 | 0 | 3+ | 62.73 | 482.98 | 36.70 | 268.89 | 54.00 | 309.00 | 28.00 | 154.00 | 76.00 | 400.00 | 30.00 | 256.00 |
| 67 | Male | 45 | 0 | 1 | 2+ | 435.48 | 109.95 | 18.06 | 335.64 | 321.00 | 78.00 | 15.00 | 167.00 | 564.00 | 89.00 | 17.00 | 234.00 |
